# A Comprehensive Review of Computer-Aided Diagnosis of Major Mental and Neurological Disorders and Suicide: A Biostatistical Perspective on Data Mining

**DOI:** 10.3390/diagnostics11030393

**Published:** 2021-02-25

**Authors:** Mahsa Mansourian, Sadaf Khademi, Hamid Reza Marateb

**Affiliations:** 1 Department of Medical Physics, School of Medicine, Isfahan University of Medical Sciences, Isfahan 81746-73461, Iran; mansourian@med.mui.ac.ir; 2 Biomedical Engineering Department, Faculty of Engineering, University of Isfahan, Isfahan 8174-67344, Iran; sadafkhademi@eng.ui.ac.ir

**Keywords:** Alzheimer’s disease, bipolar disorder, computer-aided diagnosis, data mining, dementias, depressive disorders, mental disorders, neurological disorders, schizophrenia, validation methods

## Abstract

The World Health Organization (WHO) suggests that mental disorders, neurological disorders, and suicide are growing causes of morbidity. Depressive disorders, schizophrenia, bipolar disorder, Alzheimer’s disease, and other dementias account for 1.84%, 0.60%, 0.33%, and 1.00% of total Disability Adjusted Life Years (DALYs). Furthermore, suicide, the 15th leading cause of death worldwide, could be linked to mental disorders. More than 68 computer-aided diagnosis (CAD) methods published in peer-reviewed journals from 2016 to 2021 were analyzed, among which 75% were published in the year 2018 or later. The Preferred Reporting Items for Systematic Reviews and Meta-Analyses (PRISMA) protocol was adopted to select the relevant studies. In addition to the gold standard, the sample size, neuroimaging techniques or biomarkers, validation frameworks, the classifiers, and the performance indices were analyzed. We further discussed how various performance indices are essential based on the biostatistical and data mining perspective. Moreover, critical information related to the Transparent reporting of a multivariable prediction model for individual prognosis or diagnosis (TRIPOD) guidelines was analyzed. We discussed how balancing the dataset and not using external validation could hinder the generalization of the CAD methods. We provided the list of the critical issues to consider in such studies.

## 1. Introduction

**Figure 1:**
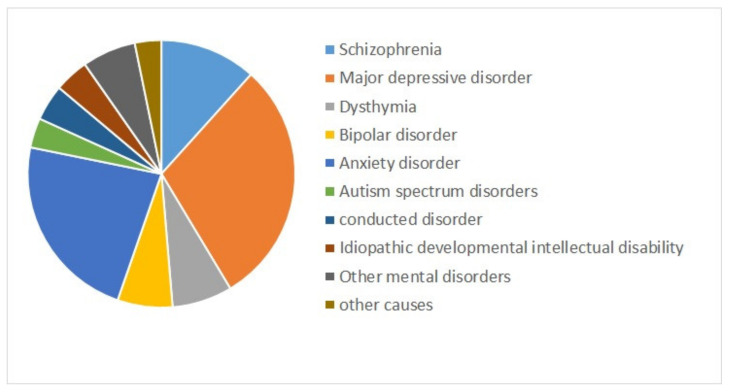
The contribution of mental disorders to Disability Adjusted Life Years (DALYs) worldwide, for both sexes combined, 2019 [6].

Mental health is a state of successful cognitive function resulting in adapting to change and coping with everyday stresses of life [1,2]. Mental disorders refer to a wide range of conditions affecting mood, thinking, and behavior. They could be occasional or chronic [3]. Some major mental disorders include depression, bipolar disorder (BD), and schizophrenia (SZ) [4]. Mental illnesses are globally among the leading causes of disability in Disability Adjusted Life Years (DALYs) [5]. Figure 1 shows the composition of mental disorder DALYs by type of disorder for both sexes combined worldwide from 1990 to 2019 [6]. Depressive disorders (29.74%), followed by anxiety disorders (22.86%), and schizophrenia (11.66%) are the top three contributors to mental disorder DALYs [6].

**Figure 2:**
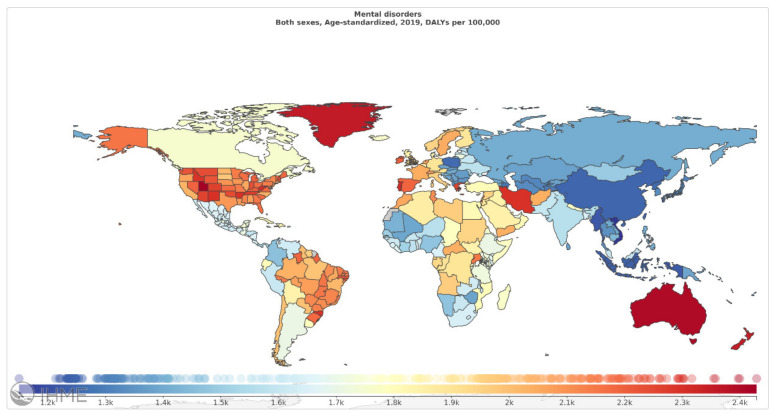
Mental disorders, age-standardized DALY rates (per 100 000) by location, both sexes combined, 2019 (reproduced with permission from [6]).

Among mental disorders, depressive disorders account for 1.84%, anxiety disorders for 1.13%, schizophrenia for 0.60%, and BD for 0.33% of total DALYs [6]. As mentioned in Figure 2 (Source: Institute for Health Metrics Evaluation. Used with permission. All rights reserved.), countries with the highest age-standardized mental disorder DALYs rates were Portugal 2603.92, Greece 2510.55, Greenland 2486.44, Iran 2436.44, and Spain 2396.768 DALYs per 100,000, in 2019 [6]. The World Health Organization (WHO) reported that over 450 million people worldwide suffer from mental disorders [7].

Every year, almost 25% of people experience a mental disorder [8]. However, due to the lack of access to adequate mental illness services and stigmatization, most patients do not receive help [9]. The increasing rate of mental disorders could be related to political and social violence, economic change, and cultural disruptions [10].

**Figure 3:**
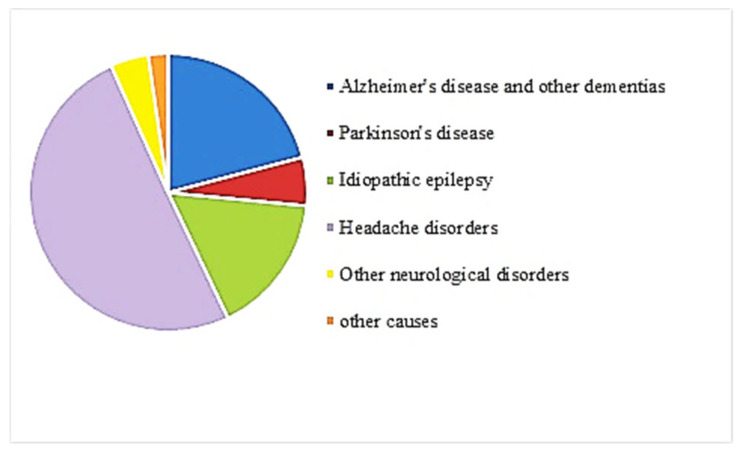
Contribution by neurological disorders to DALYs worldwide, both sexes combined, 2019 [6].

In addition to mental disorders, neurological disorders are illnesses causing psychological symptoms [11]. Such disorders have become important causes of death and disability worldwide [12]. The primary neurological disorders include Alzheimer’s disease (AD) and other dementias [12]. Figure 3 shows the composition of neurological disorders DALYs by type of disorder for both sexes combined worldwide from 1990 to 2019 [6]. About 20% of neurological disorders are AD and other dementias [13]. Today, almost 35.6 million people suffer from AD worldwide. This number will approximately double to 65.7 million cases by 2030 and may even triple to 115.4 million cases by 2050 [14]. The rapidly growing potential of sufferers and the inevitable enormous economic effects of AD on health and social services have led governments to take swift action to eradicate the disease [15]. Therefore, although AD is not at the top in Figure 3, it could be one of the most critical neurological disorders.

**Figure 4:**
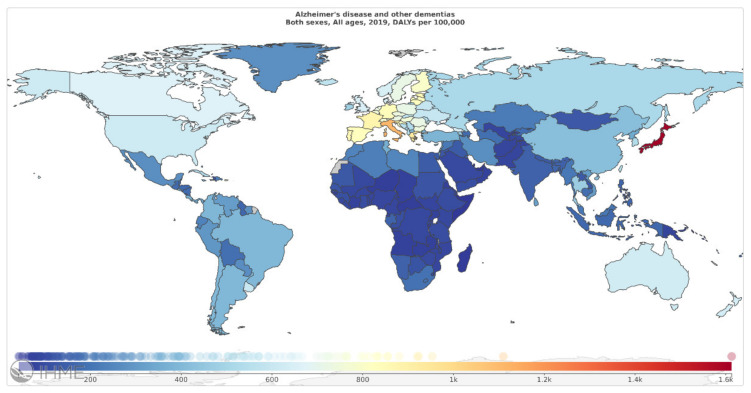
Alzheimer’s disease and other dementias, age-standardized DALY rates (per 100 000) by location, both sexes combined, 2019 (reproduced with permission from [6]).

According to the Global Burden of Disease (GBD) 2019, AD and other dementias account for 1% of total DALYs. As mentioned in Figure 4 (Source: Institute for Health Metrics Evaluation. Used with permission. All rights reserved.), countries with the highest age-standardized neurological disorders DALYs rates were: Japan 1612.77, Italy 1109.73, Greece 923.58, France 880.49, and Estonia 854.71 DALYs per 100,000, in 2019 [6].

Suicide, a death caused by intentional termination of one’s own life, has been known to be a critical public health issue by the WHO [16]. Each year, around one million people die due to suicide [17]. It is also one of the leading causes of death among young people worldwide, and, as such, it is responsible for a massive amount of pointless suffering and a substantial number of premature deaths [18]. Suicide has disruptive psychosocial effects [18] and is thus a global public health issue [19]. It shows considerable differences between geographic regions, socio-political realities, age groups, and genders [19]. Suicide was in the leading ten causes of death in five GBD regions [20].

The WHO data suggests that mental disorders, neurological disorders, and suicide are growing causes of morbidity [16,21]. The World Health Report 2001 and the Mental Health Action Plan 2013–2020 focused on mental disorders such as depression and schizophrenia, some neurological disorders like AD [22], and suicide [16]. In 2017, mental disorders were the sixth leading cause of DALYs and the second leading cause of disease burden in terms of years lived with disability (YLDs) in the world [23]. Furthermore, neurological disorders ranked as the second-leading cause of death and DALYs’ major cause in 2015 [12]. Suicide is the 15th leading cause of death worldwide [24]. Meanwhile, the total number of deaths from suicide increased by 6.7% globally from 1990 to 2016 [20]. It is also considered the second cause of unnatural death for those between 15 and 29 years old [25,26].

Significant proportions of mental and neurological disorders arise in low- and middle-income countries [27,28]. Mental disorders lead to significant social, personal, and economic loss, including functional impairment, psychosocial disability [29], low quality of life [30], and loss of productivity [31]. Patients with mental disorders have a shorter life expectancy than the general population; there is a strong dose–response effect between mortality and psychological distress [32]. Furthermore, milder disorders could impair functional capacity, which causes difficulties in social and marital relations [33].

Although in low-income and middle-income countries, 75·5% of deaths by suicide occur, suicide’s prevalence is higher in high-income countries [24]. Suicide could be linked to mental disorders [34]. Almost 90% of individuals who committed suicide have been subjected, at least, to one mental disorder [35]. Mental disorders contribute between 47% and 74% of suicide risks [18]. In around 50–65% of suicide cases, depression was observed [18]. Schizophrenia also accounts for very few of all youth suicides [36]. Furthermore, associations between suicide and anxiety disorders have been observed [18]. Accordingly, suicide prediction and diagnosis were also analyzed in our study.

Failure to detect mental disorders results in not receiving potentially effective treatment for the patients [32]. Long-lasting psychological distress has profound effects on the prospect of having a reasonable quality of life in patients and their work capacity and family [32]. It has been shown that early detection of mental disorders could shorten the duration of a disorder, reduce the number of further consultations, and result in less social impairment [32]. Furthermore, early detection of neurological disorders is critical to achieve optimal disease control [37].

There are various methods to detect and diagnose mental and neurological disorders at early stages, from interpreting participants’ answers to questions about their lives to using diagnostic equipment such as electroencephalogram (EEG), magnetoencephalogram (MEG), positron emission tomography (PET), magnetic resonance imaging (MRI), etc. [38,39]. However, manual assessment of such techniques is time-consuming and sensitive to error [39]. In fact, because of the differences in experts’ experience, manual methods of diagnosis are subjective to the examiner and are thus prone to errors and biases. Computer-aided diagnosis (CAD) was recently used as the second opinion to assist the diagnosis procedure [39].

Machine learning methods, with the inputs from different sources such as functional MRI (fMRI) [40], clinical and sociodemographic variables [41], information posted on social networks [26], or Patient Health and other related Questionnaire [42], were used in the literature for suicide diagnosis and prediction. CAD systems have been used to help clinicians, medical doctors, or neurologists diagnose certain diseases or disorders [43]. CAD systems’ goal is to improve the accuracy of experts interpreting big medical data so that the analysis time can be reduced and the diagnosis consistency is improved [44]. Numerous CAD frameworks and methods have been developed in the literature to analyze medical signals and images [43]. CAD systems are suitable to complete the neuropsychological assessments conducted by expert clinicians and improve prediction accuracy. In this sense, many studies used the CAD system to detect mental disorders, neurological disorders, and suicide. Thus, this review aimed to analyze the current CAD method for diagnosing depressive disorders, BD, schizophrenia, AD, dementia, and suicide.

## 2. Materials and Methods

### 2.1. Gold Standard

Due to the multiplicity of mental disorders and the importance of proper diagnosis and treatment, the need to classify these disorders has always existed and led to the publication of the Diagnostic and Statistical Manual of Mental Disorders (DSM). Its latest version, DSM-5, was released in 2013. Structured Clinical Interview for DSM-5 (SCID-5) is a structured diagnostic interview to diagnose mental disorders according to the criteria characterized in the DSM-5, which a trained clinician should prescribe. This structure specifies the order of the questions, how the questions are worded, and how the subject’s responses are classified. The primary diagnosis methods are summarized as the following [45].

#### 2.1.1. Depression Disorder

SCID is considered to be the commonly used gold standard for a depression diagnosis. Major depressive disorder (MDD) is a type of depression characterized by separate episodes of at least 14 days. Critical symptoms of MDD are depressed mood, loss of interest, weight loss or weight gain without any particular diet, insomnia or hypersomnia, frequent thoughts of death or suicide, decreased ability to concentrate and think, feelings of being worthless and guilty, psychomotor agitation or retardation, feelings of energy loss and indecisiveness. Five or more of the above symptoms, when at least one of them is one of the first two symptoms is required for a depression diagnosis [46]

#### 2.1.2. Bipolar Disorder

SCID is used as the gold standard among diagnostic interviews, but its validity will not be known until the discovery of related biomarkers. At least one period of mania is necessary for a specific diagnosis of bipolar disorder I (BD-I), while one hypomania and major depressive episode without a manic episode is essential for bipolar II (BD-II) diagnosis [47,48]

#### 2.1.3. Schizophrenia

Patients’ description of symptoms, mental state tests, and behavioral observations help psychiatrists diagnose schizophrenia based on DSM-5 criteria, which is the gold standard of diagnosis to date. The most important symptoms are delusions, hallucinations, disorganized speech, extremely catatonic behavior, and negative symptoms such as decreased emotional expression. Two or more of these symptoms, when at least one of them is one of the first three symptoms is required for a schizophrenia diagnosis, and each of them should be present for a considerable period within a month [49,50].

#### 2.1.4. Alzheimer’s

AD is a specific type of dementia. The gold standard hallmarks for definitive diagnosis of AD are cortical atrophy, amyloid-predominant neuritic plaques, and tau-predominant neurofibrillary tangles validated by postmortem histopathological examination. Amyloid precursor protein (APP), presenilin 1 (PSENl), or presenilin 2 (PSEN2) are known causative genes of the AD where genetic tests can show their mutation in early-onset cases. Furthermore, amyloid-based diagnostic tests such as positron emission tomography (PET) and cerebrospinal fluid (CSF) scans can be useful diagnostic tools [51]

#### 2.1.5. Dementia

In DSM-5, major neurocognitive disorder (MCD) is considered an alternative term for dementia that was used in previous versions. A significant decrease in the level of the subject’s cognitive performance; for example, in learning and memory functions, followed by interference with independent daily activities, is a sign of dementia. Clinical Dementia Rating (CDR) is a cognitive diagnostic assessment widely used as the gold standard for diagnosing dementia. The CDR test is a semi-structured interview with the patient and a trustful informant, consisting of 46 questions, that takes 30–90 min to be completed and must be done by a trained clinician [52,53,54].

#### 2.1.6. Suicide

Validated questionnaires have been used in the literature to diagnose high-risk individuals for suicidal behaviors [55]. Suicide Behaviors Questionnaire-Revised (SBQ-R) is a globalized test for identifying individuals at increased risk of suicidal behaviors, including ideation and attempts [56]. The SBQ-R test was designed based on the SBQ test, a 34-item questionnaire measuring the suicide tendency. It is a self-report test distinguishing between suicidal and non-suicidal subjects. The SBQ-R test includes four Likert-type questions that measure the risk of suicide according to the subject’s suicide ideation/attempt during lifetime, suicidal ideation rate in the last year, expressing thoughts of committing suicide with others, and suicidal behavior occurrence probability in the future. Each question has different points from 0 to 6 based on the subject’s choice. Two scoring criteria have been proposed so far to classify suicidal and non-suicidal individuals based on SBQ-R results: SBQ-R Item 1 and SBQ-R total score varying between 3 and 18. Clinical and non-clinical samples have an identical cutoff score of 2 in the SBQ-R Item 1. The SBQ-R total score’s cutoff scores were 7 and 8 for clinical and non-clinical samples, respectively [42].

### 2.2. The Literature Review

There are currently not enough biomarkers in psychiatry to classify disease state from the normal state, so diagnosis mostly depends on patient–physician interactions and questionnaires. Clinical observations based on patient self-reports are subjective and inaccurate even if they are based on DSM-5 criteria since they cannot identify false positives and recognize disorders from risks. This is where artificial intelligence (AI) comes in handy. AI is a general term in psychiatry that denotes the use of advanced computerized techniques and algorithms to diagnose, prevent, and treat mental disorders, such as automatic speech processing and machine learning algorithms applied on electronic medical databases and health records to assess a patient’s mental state. AI-based interventions reduce false negative and positive diagnoses and annihilate the stigma associated with mental illness symptoms to the clinician. They are also affordable and have significant benefits for patients suffering from restricted movement due to their symptoms. AI-based methods are not replacing clinicians; they can complement human clinical decisions by providing more comprehensive information to empower the health care system [57,58]. Here, we provided the literature review of the CAD systems for suicide, neurological disorders, and mental disorders focusing on the sample size, input features, classifiers, type of validations, and their performance indices.

#### 2.2.1. PRISMA Guideline

We reviewed the works focusing on the diagnosis and prediction of CAD methods proposed in the literature for suicide, neurological disorders, and mental disorders. The Preferred Reporting Items for Systematic Reviews and Meta-Analyses (PRISMA) statement [59,60] was proposed in the literature to enrich and standardize medical reviewer papers [61]. We adopted the PRISMA guideline to select the relevant studies.

#### 2.2.2. Search Strategy

A literature search of the online database of PubMed between 2016 and 2021 was performed using the terms (“bipolar” OR “bipolar disorder” OR “schizophrenia” OR “suicide” OR “Alzheimer” OR “dementia” OR “major depressive disorder” OR “depression”) AND (“machine learning” OR “deep learning”) AND “accuracy”. The reference lists of the identified publications were also reviewed. Peer-reviewed articles in English on Humans were analyzed.

#### 2.2.3. Eligibility Criteria

**Figure 5:**
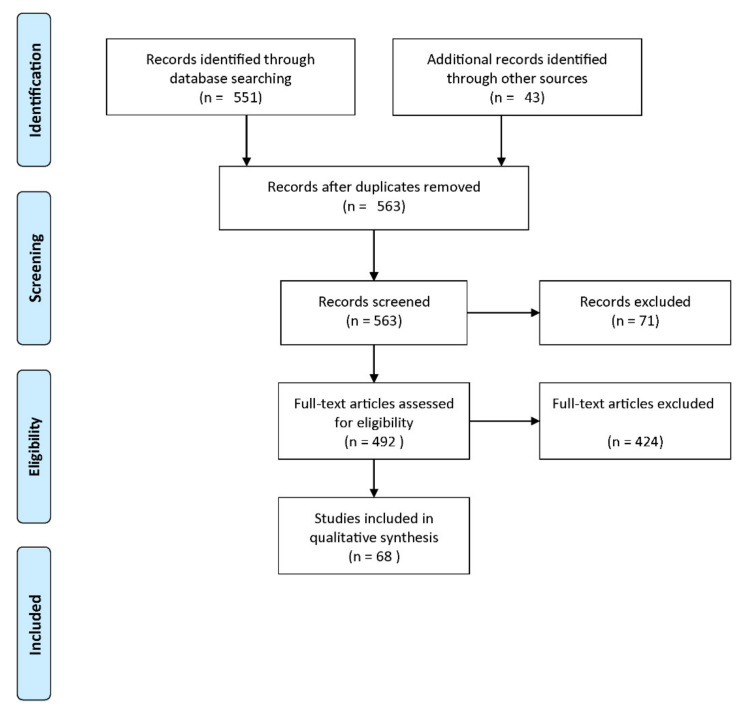
Flow diagram of the study selection process (reproduced with permission from [60]).

Published studies were included in the review if they met the following criteria: (1) at least a measure of the diagnostic accuracy was provided, (2) at least the classifier, the validation framework, or the validation type were provided. Figure 5 shows a flow diagram describing the study selection process. Among 563 records screened, 71 studies were excluded as irrelevant to the original research question. Among the remaining 492 studies, 424 studies did not meet the eligibility criteria. Thus, 68 studies were included in our analysis.

#### 2.2.4. Data Abstraction

The following characteristics were recorded for each study included in our analysis: publication reference (the first author’s surname and the year of publication), the sample size, the case and control groups, input features, classifiers, internal or external validation, type of validation (holdout or resampling), and the diagnostic accuracy.

## 3. Results

The CAD methods for mental and neurological disorders are listed in Table 1, Table 2, Table 3, Table 4, Table 5, Table 6, Table 7, while the CAD methods for suicide prediction are provided in Table 8, Table 9, Table 10, Table 11.

### 3.1. Validation Frameworks and Performance Indices

#### 3.1.1. Validation Frameworks

The validation framework is one of the critical issues in data mining approaches. In “holdout,” the most straightforward cross-validation, the data set is randomly assigned to two sets: the training set and the test set. In addition to the data’s inefficient use, the method’s limitations are pessimistically biased error estimations [127,128]. Moreover, testing hypotheses proposed by the data are not guarded by this method (type III errors [129]) as the data may be permuted until there would be an acceptable accuracy on the training and test sets in a “holdout” setting. Therefore, other validation frameworks such as repeated holdout, leave-one-out validation, 0.632+ bootstrap, and cross-validation [130] are preferred. These issues are also addressed in the TRIPOD guideline from a clinical perspective [131].

Choi et al. [104] proposed a framework for early detection of dementia using holdout validation. Moreira et al. [105] presented a hybrid data mining model for the diagnosis of dementia using holdout setting. Lin et al. [97] designed a convolutional neural network (CNN)-based approach to predict mild cognitive impairment to Alzheimer’s disease (MCI-to-AD) conversion using MRI data with leave-one-out cross-validation (CV). Ding et al. [98] proposed a hybrid computational approach to classify AD with holdout and resampling; synthetic minority oversampling technique (SMOTE). Aidos et al. [101] presented a new methodology to obtain an efficient CAD system for predicting AD using longitudinal information with holdout validation. Li et al. [132] developed a spectral CNN for a reliable AD prediction with 10-fold CV. Sayed et al. [133] designed an automatic system for AD diagnosis with 7-fold CV.

#### 3.1.2. Subject-Wise Cross-Validation

The other critical issue is using leave-one-subject-out cross-validation when there are repeated measurements for each subject [134]. Thus, we must take out the entire measurements of a subject from the training set and report the trained system’s performance for the test subject. Otherwise, if we use other internal validation methods and perform training and test set random permutations on the entire measurements, rather than subjects, the probability of some measurements of one subject being in the training set and others in the test set is high. If there is a high correlation in such repeated measurements, the accuracy of the diagnosis system is overestimated. To reduce estimation variance, it is preferred to use subject-wise cross-validation with a more extensive test sample size, rather than leave-one-subject-out cross-validation [135].

#### 3.1.3. Critical Performance Indices

It is also essential to report various performance indices since they convey critical information that is very important in clinical systems. One of the most important formulas related to the posterior probability is the following [136]:

(1) $$PPV=P\left(D\backslash E\right)=\frac{Se\times Prev}{Se\times Prev+\left(1-Sp\right)\times \left(1-Prev\right)}$$

where, *Se* is the sensitivity, *Sp* is the specificity, *Prev* is the prevalence of the disease, *D* is the positive condition event determined by the gold standard, and *E* is the test outcome positive event determined by the diagnosis system. The parameter *PPV* is the disease probability given that the patient test result is positive, which is essential when the system is used in practice. The *PPV* significantly drops in imbalanced datasets, in which the prevalence of the disease is low. For example, when a CAD with the *Se* and *Sp* of 80% and 95% is tested in practice where *Prev* is 10%, the expected *PPV* is 64%.

The minimum sensitivity of 80% and specificity of 95% [137], maximum False Discovery Rate (FDR = 1-PPV, Positive Predictive Value) of 5% [138], and the minimum Diagnostic Odds Ratio (DOR) of 100 [139] could be considered a reasonable requirement of a reliable clinical diagnosis system. As a complementary condition, the minimum Negative Predictive Value (NPV) of 95% could be listed [136].

Some of the published works on mental health provided a variety of performance indices. For example, Lee et al. [62] designed a diagnostic model using biomarkers in peripheral blood to diagnose BD-II with a 90% specificity and sensitivity of 85%. Ildiz et al. [73] obtained 94% sensitivity, specificity, and precision of their analytical model to diagnose SZ and BD. Alici et al. [63] proposed the utility of optical coherence tomography (OCT) data to distinguish BD-I patients from controls with a sensitivity of 87.5%, a specificity of 47.5%, positive predictive value (PPV) of 52.5%, and negative predictive value (NPV) of 79.2%. Fernandes et al. [66] reached a sensitivity of 88.29% and specificity of 71.11% for BD vs. control, a sensitivity of 84% and specificity of 81% for SZ vs. control, and sensitivity of 71% and specificity of 73% for BD and SZ. Achalia et al. [74] used multimodal neuroimaging and neurocognitive measures to differentiate BD patients from healthy controls and obtained a sensitivity of 82.3% and specificity of 92.7%. Li et al. [140] obtained a sensitivity of 80.6% and specificity of 86.3% in predicting AD with Actigraphy Data. Li et al. [132] showed that their spectral CNN could achieve a sensitivity of 88.24% and specificity of 95.45% in AD and normal control classification, a sensitivity of 92.86% and specificity of 77.78% in AD and MCI classification, and sensitivity of 84.38% and specificity of 92% in MCI and normal control classification.

A machine learning approach was used by Bin-Hezam and Ward [102] to detect dementia and yielded a precision of 91.34%, a sensitivity of 91.53%, and F1 score of 91.41% for dementia vs. non-dementia, a precision of 76.76%, sensitivity of 77.00%, and F1 score of 76.35% for control normal (CN) vs. MCI vs. dementia. Choi et al. [104] proposed a novel framework for dementia identification with an F1 score of 78%, sensitivity of 93.43%, specificity of 89.66%, positive likelihood ratio of 9.0319, a negative likelihood ratio of 0.0732, PPV of 0.5064, and NPV of 0.9917. Chen et al. [117] used ensemble learning to predict suicide attempts/death following a visit to psychiatric specialty care. The sensitivity, specificity, PPV, and NPV of the 90-day prediction model were 47.2%, 96.6%, 34.9%, and 97.9%. Ensemble learning was also used by Naghavi et al. [42] for the prediction of suicide ideation/behavior. The proposed system had the sensitivity, specificity, PPV, and DOR of 81%, 98%, 94%, and 227, respectively. In such examples, various performance indices could provide valuable information about the designed systems’ clinical reliability. Otherwise, it is not possible to judge the clinical applications of CAD systems.

#### 3.1.4. The 95% Confidence Interval

Following the STARD and TRIPOD guidelines, it is necessary to provide the confidence interval (CI) 95% of the performance indices [141,142]. Such CI 95% values could identify the reliability of the performance indices estimation [143]. For example, in the study by Shang-Ming Zhou et al. [103], effective predictors related to hospital admission of dementia patients such as blood glucose were found with a sensitivity of 0.758 (95% CI 0.731–0.785), specificity of 0.759 (95% CI 0.710–0.808), precision of 0.766 (95% CI 0.735–0.797), and negative predictive value of 0.751 (95% CI 0.741–0.761). Xuemei Ding et al. [98] achieved a multiclass accuracy of 0.8 (95% CI 0.67–0.89) to classify Alzheimer’s disease severity. Kelvin KF Tsoi et al. [144] showed that the combination of drawing behavioral data and digital platform could be useful in early detection of dementia with a sensitivity of 0.742 (95% CI 0.702–0.779), specificity of 0.724 (95% CI 0.668–0.776), positive predictive value of 0.833 (95% CI 0.804–0.859), and negative predictive value of 0.601 (95% CI 0.562–0.640).

Klaus Munkholm et al. [70] demonstrated that a composite marker containing different molecular levels and tissue data is an operational biomarker to discriminate bipolar disorder from healthy subjects with an Area Under the ROC Curve (AUC) of 0.826 (95% CI 0.749–0.904). Utilizing optical coherence tomography, Soner Alici et al. [64] indicated an AUC of 0.688 (95% CI 0.604–0.771) in comparing bipolar disorder and healthy individuals. In 2016, Guoqing Zhao et al. [64] performed a study and mentioned that plasma mBDNF and proBDNF levels were the best biomarkers in identifying bipolar disorder among patients in depressive episodes with an AUC of 0.858 (95% CI 0.753–0.963). In the study by Noa Tsujii et al. [67], a high AUC of 0.917 (95% CI 0.849–0.985) was provided based on hemodynamic response and mitochondrial dysfunction to diagnose bipolar disorder and major depressive disorder. Naghavi et al. [42] assessed the suicide ideation/behavior performance using different indices and CI 95%. Based on the cross-validated confusion matrix, the AUC, Matthews Correlation Coefficient (MCC), Discriminative Factor (DP), and Cohen’s Kappa were 0.90 (CI 95%: 0.86–0.93), 0.83 (0.81–0.86), 227 (100–512), 2.30 (1.96–2.65), and 0.83 (0.78–0.88). Chen et al. [117] predicted the suicide attempt/death with an AUC of 0.88 (0.87–0.89) for the outcome within 90 days.

### 3.2. Input Features

Various inputs were used in the literature for mental and neurological disorder diagnosis. They include, for example, Child Behavior Checklist [145], serum miRNA [62], blood serum Raman spectra [73], optical coherence tomography [63], blood samples [64,65], immune and inflammatory biomarkers in peripheral blood and cognitive biomarkers [66], blood sample Nuclear Magnetic Resonance (NMR) [69], optical coherence tomography [64], MRI [76,82], fMRI [103,114,118], rs-fMRI [72,86], PET [96], EEG [79,81], steady-state visual evoked potentials (SSVEP) [71], speech signal [86], demographics and medical history [102], or drawing behavior [144].

Moreover, demographic, socioeconomic and medical records [109], fMRI [40], Weibo posts [109], questionnaire and web-based survey [40], and Reddit social media dataset [126] were used to predict or diagnose suicide ideation, behavior, or death.

Functional neuroimaging techniques—such as PET and fMRI—enable mapping the brain’s physiology by measuring blood flow, receptor–ligand binding, and metabolism. Such techniques have been recently used in mental health, which improved understanding of the underlying mechanisms [146]. Functional imaging is divided into resting state (e.g., rs-fMRI) and studies in active conditions. On the other hand, structural neuroimaging, such as NMR and MRI, has been widely used to exclude organic brain disease in mental disorders. It was shown in the literature that structural brain imaging is clinically useful to discriminate mental disorders, including SZ, BD, depression (MDD), and AD [147].

Both of the functional and structural—except CT-scan—neuroimaging techniques were shown to be useful for suicided diagnosis [148]. Both techniques have advantages and disadvantages (e.g., spatial versus temporal resolution) [149], and their combination, a.k.a., multimodal neuroimaging, can yield important insights due to its complementary spatiotemporal resolution [150]. Lei et al. used the combination of MRI and rs-fMRI for diagnosing SZ patients. In this study, the multimodal neuroimaging showed better performance than structural or functional neuroimaging separately [151].

**Figure 6:**
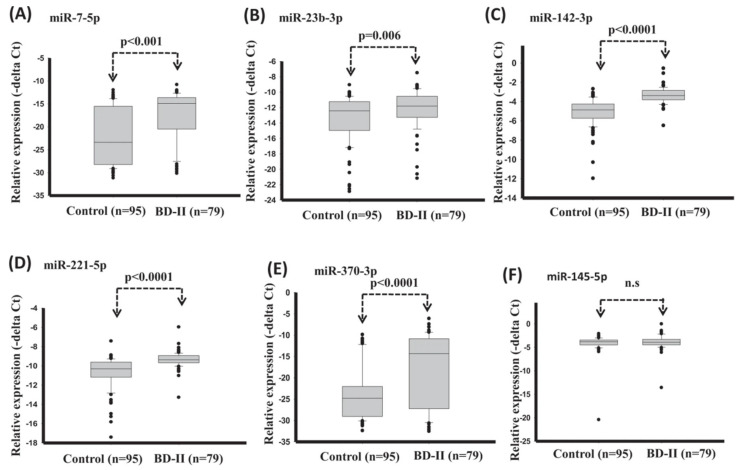
Expression levels of circulating miRNAs in serum in healthy controls and BD-II using *t*-test (training set). (**A**) miR-7-5p (**B**) miR-23b-3p (**C**) miR-142-3p (**D**) miR-221-5p (**E**) miR-370-3p (**F**) miR-145-5p. (Reproduced with permission from [62]).

A promising feature for the BD-II diagnosis was introduced by Lee et al. [62], which was the serum miRNA. In this study, serum expression levels of miR-7-5p, miR-23b-3p, miR-142-3p, miR-221-5p, and miR-370-3p significantly reduced in healthy control compared with BD-II (Figure 6). The diagnostic model with support vector machine (SVM) reached good diagnostic accuracy (AUC: 0.907) when using expression of miRNA miR-7-5p + miR-142-3p + miR-221-5p + miR-370-3p.

Perhaps the mostly used features for suicide ideation/attempts prediction are demographics, socioeconomic status (SES), and life-style variables. For example, Jung et al. [113] designed a suicide prediction model for middle and high school students based on the multivariate logistic regression and reached the prediction accuracy of 77.9%. The selected significant features included gender, school grade, city type, academic achievement, living with parents, family SES, father’s and mother’s education, physical activity, and self-rated weight and health.

### 3.3. Classification Methods

A variety of classification methods were used in the literature to classify mental and neurological disorders. The support vector machine (SVM) was used to diagnose BD [62]. Partial least squares discriminant analysis (PLS-DA) [66], k-nearest neighbor [71], deep convolutional neural network (CNN) [78], and Fisher linear discriminant (FLD) [86] were used for SZ classification. The multivariate logistic regression (MLR) [67], deep integrated support vector machine (DISVM) [93], CNN [94], and SVM [96] were used to classify depression. The SVM, artificial neural network (ANN), decision tree [106], and CNN [99] were used for AD/MCI diagnosis.

Many classifiers were used for suicide ideation, behavior, or death prediction in the literature, including logistic regression with/without regularization [99], deep neural networks (DNNs) [104,125], decision tree algorithm [99], SVM [40], random forests [104,125], Gaussian Naive Bayes (GNB) [40], extreme gradient boosting (XGB) [40], Cox regression [116], ensemble learning [117], elastic net [41], and long short-term memory convolutional neural network (LSTM-CNN) [126].

**Figure 7:**
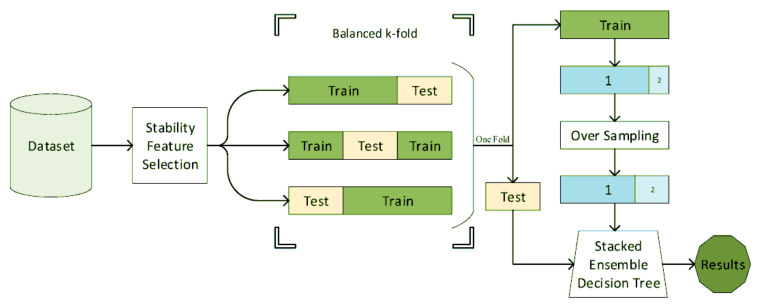
The block diagram of the suicide diagnosis algorithm. The features are first selected using stability feature selection. Using the stratified sampling, the features are then classified using a stacked ensemble decision tree (reproduced with permission from [42]).

Decision tree, or its ensemble extensions such as random forests were frequently used for mental health in the literature [42,105,106,107,108,112,118,120,122]. A decision tree is a rule-based system, wherein its simplest form is a clinically interpretable structure for clinicians used in clinical decision analysis [152]. Naghavi et al. [42] used the combination of stability feature selection and stacked ensembled decision trees (Figure 7) for suicide ideation/behavior diagnosis and reached an AUC of 0.9. In this study, a variety of questionnaires and demographic information was used.

The classifiers used for mental health could be categorized into two main categories: traditional machine learning (e.g., DA and its variants, SVM, decision tree), and deep learning (LSTM, CNN). A deep neural network (DNN) is an artificial neural network with more than one hidden layer. Unlike many traditional classifiers such as linear discriminative analysis (LDA), SVM, or Decision Tree (DT), where few parameters must be estimated or tuned, DNNs have many tunable variables. Thus, they require massive amounts of data to estimate their parameters accurately. When the available data is limited, various issues must be considered to avoid overfitting [153]. Strategies such as early stopping criteria, data augmentation, dropouts, and regularization are used [154]. Moreover, when the dataset is imbalanced (e.g., the mental disorder classification) specific deep learning techniques must be taken into account [155]. Geometrical augmentation is usually used to increase the image sample size by random rotation, translation, and horizontal flipping. However, it was shown that such augmentations do not necessarily improve the predictive accuracy of the deep learning methods [156].

DNNs were used in the literature for multimodal neuroimaging classification in mental health [157]. Although DNNs are promising, they usually appear to be black boxes. The input is the raw data, and the output is the predicted class, and no internal interpretation is provided. It is problematic since clinicians require proper interpretation of abnormal brain regions, for example, in neuroimaging data [158]. There have been some attempts to visualize the black box of the DNNs in the literature [159].

Statistical models such as MLR and Cox regressions were used in mental health literature [67,116]. MLR is an extension of the linear regression when the outcome is binary. It not only provides the probability that a sample belongs to an output class, but it also identifies the significant features in the model. Thus, it is also a feature selection method [160]. On the other hand, Cox regressions are time-to-event models where the event of interest (e.g., committing suicide) and the event’s time (e.g., the time from the suicide attempt to the previous hospitalization) are essential. Such models are usually used in survival analysis. When a proper threshold is estimated, it is possible to dichotomize the model’s continuous output risk for discrimination between output classes [161]. Unlike other classification methods, both MLR and Cox models support mixed-type input data, and no transformation is required to perform on nominal or ordinal data.

### 3.4. Balancing the Dataset and Generalization of the Results

**Figure 8:**
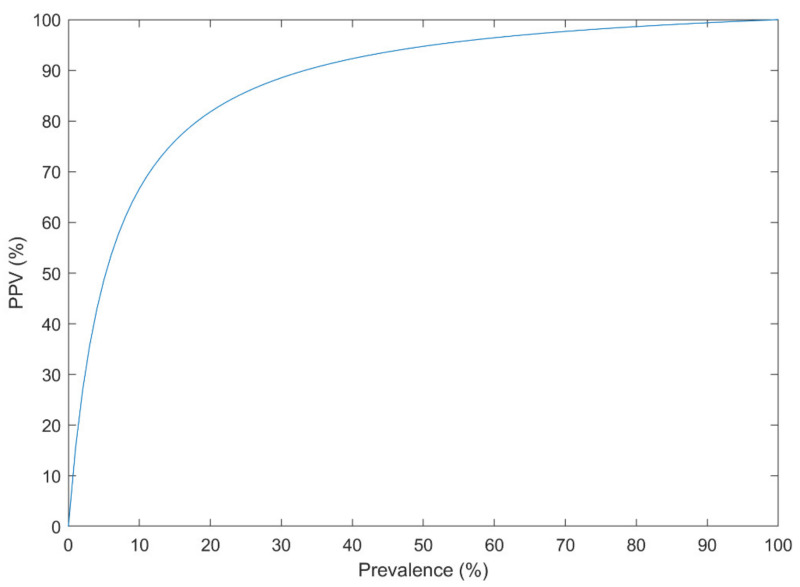
The Positive Predictive Value (PPV) of a diagnosis system with the sensitivity of 90% and specificity of 95% at different disease prevalence.

Bayes’ theorem (Equation (1)) was addressed in the literature as a confounding effect of the low prevalence of a disorder on the performance of the CAD systems [162], even when the AUC is very high [163]. Events such as suicide attempt/death have a low prevalence in the population (e.g., 10.7 per 100,000 individuals [164]). Other mental and neurological disorders have a relatively low prevalence (e.g., the global prevalence of 1% for SZ [165]). Thus, they can only be reliably predicted using an extraordinary discrimination capability between higher and lower risk groups. Suppose that a CAD system has a Sensitivity of 90% and a Specificity of 95% based on the cross-validated confusion matrix, which is very good for an imbalanced dataset. The probability that the new subject has the disorder, subject to the positive CAD result, could be estimated using Equation (1) for different disease prevalence (Figure 8).

For example, with the prevalence of 1% in such disorders, the PPV is only 15%. If the dataset is balanced for the analysis (e.g., 3549 suicide-indicative posts, versus 3652 non-suicidal posts in [126]), the PPV is 95% on the analyzed dataset. However, when the system is used in practice (the prevalence of 1%), the PPV drops down to 15%. Thus, the analyzed dataset must resemble the population. It is only preserved when proper sampling and sample size calculation is performed.

### 3.5. EEG-Based Diagnosis

Among the studies analyzed in Table 1, Table 2, Table 3, Table 4, Table 5, Table 6, Table 7, Table 8, Table 9, Table 10, Table 11, some use the EEG signal for diagnosis. In such studies, the number of EEG channels was shown in the tables. It is also necessary to report discriminative features based on the traditional frequency bands as important clinical biomarkers in such studies. It is not enough to show whether the classification system has an acceptable accuracy, as these discriminative features are very important for clinicians. The spatial distribution of such features must also be provided over the skull [166]. In EEG studies, either the resting state [166] or evoked or cognitive functions [167] were used for mental disorders.

**Figure 9:**
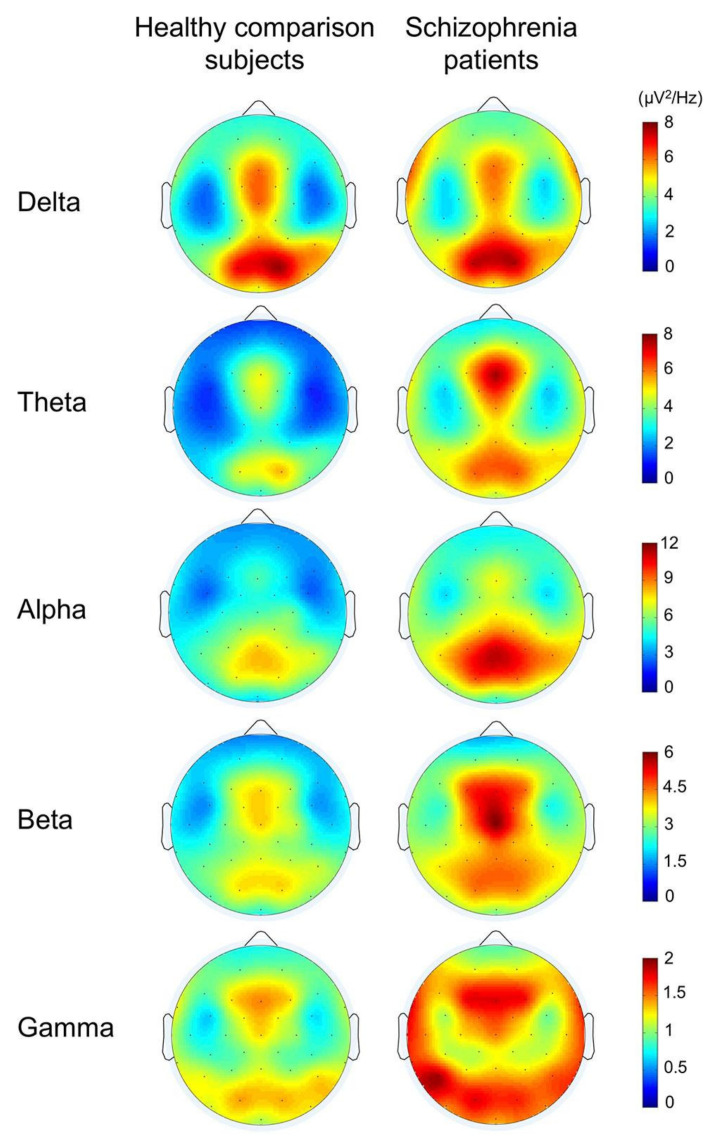
The topographies for grand average spectral power in schizophrenia patients and healthy comparison subjects (reproduced with permission from [167]).

An example was provided from the comparison between schizophrenia and healthy subjects during cognitive functions in Figure 9. It showed significantly lower power in gamma, beta, theta, and alpha bands in healthy subjects than schizophrenia patients. It also showed that more or less, it includes the entire brain. In agreement with the theory that schizophrenia is not a lesion of a part of the brain, it is a disconnection syndrome. This disconnection would be expressed in a failure to modulate synchronous activity caused by disturbances in the dopaminergic mechanism [168].

**Figure 10:**
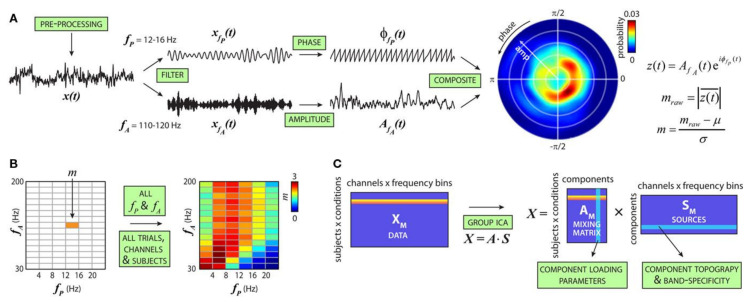
Cross-frequency modulation analysis. (**A**) Steps to compute the cfM index (m). (**B**) Steps in (**A**) are repeated for all fP and fA combinations to produce the comodulogram. (**C**) For each subject, comodulograms are averaged over trials. A single row for each condition is generated by merging data from all channels (reproduced with permission from [171]).

It is hypothesized that information flow across larger cortical networks is projected by low-frequency brain oscillations, while local cortical information processing is represented by high-frequency oscillations [169]. Thus, the interaction between different high- and low-frequency bands, also known as cross-frequency coupling (CFC) (Figure 10), could provide valuable insights into brain functions [170] and mental disorder diagnosis [171]. Such a representation is currently used instead of simple energy representation of different frequency bands. However, as the dimension increases, it is essential to select connected or disconnected regions of interest and representative interactions.

**Figure 11:**
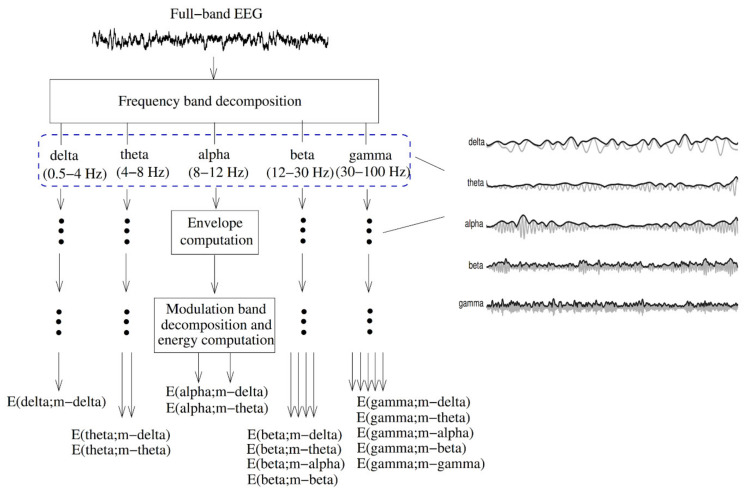
Signal processing steps used to compute resting EEG spectro-temporal modulation energy (reproduced with permission from [172]).

The EEG amplitude modulation analysis (Figure 11) has been used to diagnose AD [172]. First, the full-band EEG signal was decomposed into five sub-bands (delta, theta, alpha, beta, and gamma). The Hilbert transform was used to extract the envelope of each sub-band signal. A second frequency decomposition was then used based on modulation filters to represent cross-frequency modulation interaction [173].

**Figure 12:**
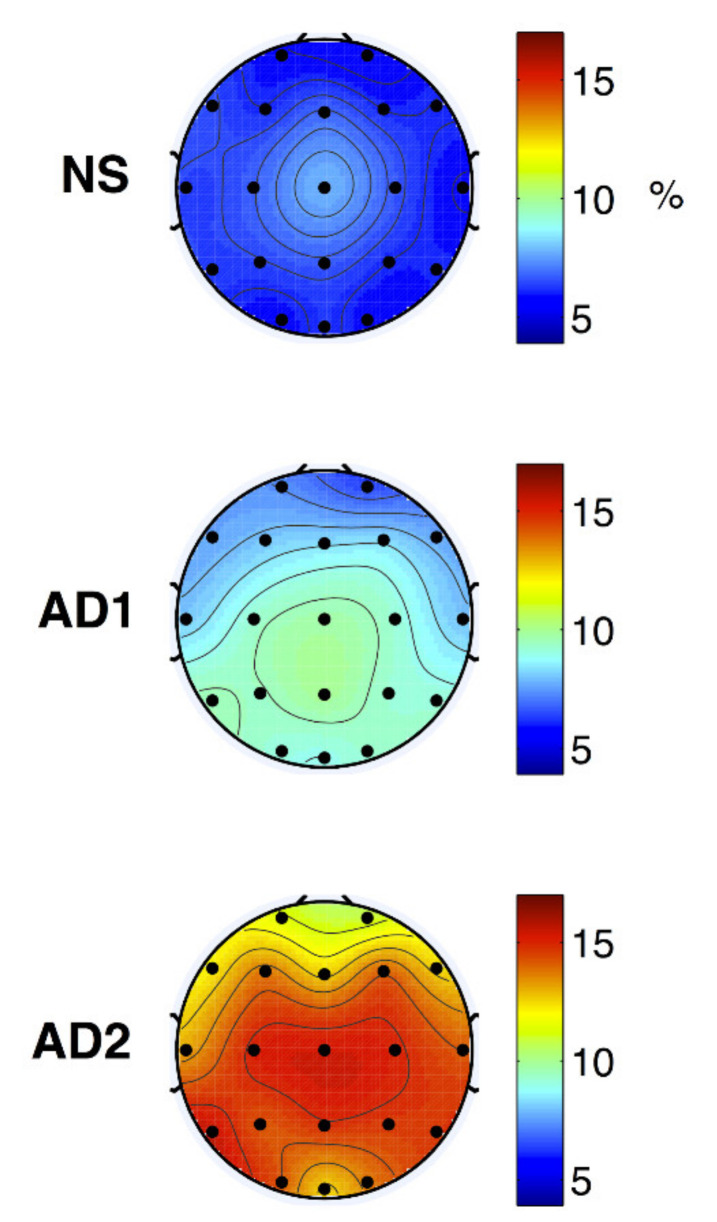
Topographical maps of average NS (top), AD1 (middle; mild AD), and AD2 (bottom; moderate AD) modulation frequency responses (reproduced with permission from [172]).

The modulation frequency bands were shown as m-delta (0.5–4 Hz) or m-theta (4–8 Hz). The m-delta modulation frequency content in the theta frequency band could discriminate between the healthy normal, mild, and moderate AD (Figure 12).

## 4. Discussion

This review focused on the data mining methods proposed in the literature to classify major mental and neurological disorders, namely SZ, BD, MDD, AD, suicide ideation, attempt, or death. More than 68 recently peer-reviewed published journal papers since 2016 were considered, among which 75% were published in the year 2018 or later. Alonso et al. [174] provided a systematic review of the major mental and neurological disorders. However, they analyzed papers published by 2017, and the data mining validation frameworks and methods focused on in our study were not covered in their study.

Moreover, other (systematic) reviews were published in the literature on this topic [175]. Jo et al. [153] analyzed deep learning papers on AD diagnosis and prognosis published between January 2013 and July 2018 in which neuroimaging data were used. Librenza-Garcia et al. [176] analyzed machine learning papers on BD diagnosis, personalized treatment, and prognosis published up to January 2017. de Filippis et al. [177] analyzed machine learning methods for structural and functional MRI SZ diagnosis published between 2012 and 2019. Castillo-Sánchez et al. [26] reviewed machine learning methods for suicide risk assessment on social networks from 2010 until December 2019. Although the classifiers, sample size, input features, and their performance were taken into account in such studies, the validation type and framework were not directly analyzed. In addition to not following the related clinical standards such as STARD and TRIPOD, these issues would avoid the widespread application of machine learning methods in practice.

Our study has some limitations. First, we only considered PubMed for the search strategy. Other online databases such as ISI, Embase, Google Scholar, and Cochrane Collaboration could improve our initial screening records. We only focused on SZ, BD, depression (MDD), AD, dementia, and suicide. Other significant disorders, including anxiety and headache were not considered. Moreover, we mainly focused on the validation type and framework with the biostatistical perspective. However, feature extraction, selection, and classifiers are essential issues in machine learning.

In our study, the epidemiological information from the GBD was provided to identify the importance of such disorders, and the gold standard methods for their diagnosis were briefly reviewed. The CAD systems were classified based on the classification goal, sample size, neuroimaging techniques, the number of channels (in EEG signals), type of validation in terms of internal and external (subject-based) methods, type of validation based on holdout, cross-validation, and resampling methods, the performance index, and its value. We also discussed the importance of reporting a variety of performance indices and their CI 95%. Some frequency–domain features used in the literature were reviewed for major mental and neurological disorders.

Some issues must be taken into account for better clinical applications of the CAD systems in this field [136]. A simple and intuitive method must present the classification features’ discrimination over the recording electrodes and (or) their interactions. The system must be validated using proper performance indices and statistical tests. The proposed system’s clinical reliability must also be identified based on Type I, II, and III errors. The clinical interpretation, using the activity maps (for example), must be provided. The rule-based systems or interaction networks are preferred over black box methods to facilitate clinical interpretation and validation [178]. Standardization (e.g., in terms of the brain frequency bands) and benchmark datasets could facilitate the comparison of the state-of-the-art and thus improve the CAD systems’ effectiveness to diagnose major mental disorders, neurological disorders, and suicide.

## 5. Conclusions

The following issues must be taken into account to improve the clinical application of the CAD systems for mental health:

- The related standards, including STARD and TRIPOD, must be used. TRIPOD-Artificial intelligence (AI) is now underway due to AI applications in CAD [179,180].
- Proper performance indices must be provided in addition to their interpretation. This issue is especially critical when the database is imbalanced, and some indices could be biased [136].
- The CI 95% of the performance indices must be provided. It is especially critical for the AUC. If its CI 95% includes 0.5, the diagnostic method’s performance is not significantly better than a random generator.
- The prevalence of the disorder in the analyzed dataset must resemble its actual prevalence in the population. Otherwise, the performance of the method in practice, a.k.a. PPV, is highly deteriorated.
- A proper validation framework must be used to avoid Type III error. External validation is the best method to improve the generalization of the designed CAD.
- The clinical interpretation of the input features, their ranking, and the classifier structure must be provided for clinicians.

## Figures and Tables

**Table 1:** CAD methods for mental and neurological disorders.

References	Goal	Sample Size	Data	Classifier	Internal, External, Validation	Type of Validation	Performance Indices
Lee et al. (2020) [62]	BD-II	(BD-II: *n* = 20, C: *n* = 20	Blood sample, Serum miRNA	Support vector machine (SVM)	Internal	Holdout	AUC: 0.91
Alici et al. (2019) [63]	BD	BD = 80, C = 80	Optical coherence tomography	logistic regression analysis	-	-	AUC: 0.69
Zhao et al. (2016) [64]	major depressive disorder (MDD) and BD	C = 44, MDD = 37 BD = 24	Blood sample	logistic regression	-	-	AUC: 0.86
Haenisch et al. (2016) [65]	BD	C = 44 l, BD = 66 (validation) Test: (First-onset MDD = 90, un-diagnosed BD = 12, C = 184 Pre-diagnostic = 110)	Blood sample	lasso regression	Both	10-fold CV	AUC: 0.8 (BD vs. first onset MDD), AUC: 0.79 (BD vs. C)
Fernandes et al. (2020) [66]	BD or SZ	blood-based domain = 323 (BD = 121, SZ = 71, C = 131), cognitive domain = 372 (SZ = 84, C = 171), multi-domain composed by the immune blood-based domain plus the cognitive domain = 279 (BD = 98, SZ = 5, C = 123)	peripheral blood sample cognitive biomarkers	linear discriminative analysis (LDA)	Internal	10-fold CV	(BD vs. C) Accuracy: 80, AUC: 0.86 (SZ vs. C) Accuracy: 86.18, AUC: 0.89 (BD vs. SZ) Accuracy: 76.43, AUC: 0.80
Tsujii et al. (2019) [67]	Distinguishing BD and MDD	58 healthy C: 58 BD: 79 MDD: 44	Blood sample, NIRS	Logistic Regression Analysis	-	-	AUC: 0.92
Faurholt-Jepsen et al. (2019) [68]	BD	BD (Euthymia, Depression, Mania): 29, C: 37	objective smartphone data reflecting behavioral activities	Gradient boosting	Internal	10-fold CV (random oversampling, sampling the minority class with replacement)	AUC: 0.66

C: (healthy) control; BD: Bipolar Disorder; SZ: Schizophrenia; MDD: Major Depressive Disorder; CV: Cross-Validation; AUC: Area Under the ROC Curve.

**Table 2:** CAD methods for mental and neurological disorders (cont’d).

References	Goal	Sample Size	Data	Classifier	Internal, External, Validation	Type of Validation	Index (the Best Result)
Tasic et al. (2019) [69]	diagnosis between SZ and BD	SZ = 54 euthymic outpatients with BD type 1 (BD) = 68, C = 60	blood serum samples; NMR	PLS-DA (Partial Least Squares Discriminant Analysis)	Internal	CV	AUC: 1 (SZ and HC), AUC: 0.87 (BD and HC), AUC: 9.93 (SZ and BD)
Munkholm et al. (2019) [70]	diagnostic test in BD	BD = 33, C = 35	blood and urine sample (211 sample, 140 BD, 71 C)	composite biomarker score	Internal	Holdout (50%)	AUC: 0. 95
Alimardani et al. (2018) [71]	Classification of BD and SZ	SZ = 23 BD = 23	SSVEP (number of channels = 21)	k-nearest neighbor	Internal	Leave one-out CV	accuracy: 91.30%
Wang et al. (2020) [72]	Classification of Unmedicated BD	unmedicated BD II = 90 C = 117	rs-fMRI	Support vector machine (SVM)	Internal	Holdout Train (BD (*n* = 72), HC (*n* = 94)) Test (BD (*n* = 18), C (*n* = 23))	accuracy: 80% AUC: 0.838
Ogruc Ildiz et al. (2020) [73]	schizophrenia (SZ) and phases of BD	40 to manic episode (BD-M) and depressive episode (BD-D), 60 to the SZ, euthymic (BD-E), C	blood serum Raman spectra	Partial Least Squares Discriminant Analysis (PLS-DA)	Internal	holdout	Accuracy: 99%
Achalia et al. (2020) [74]	BD	Type I BD = 30 HC = 30	T1 weighted three-dimensional MR images and rs-fMRI	Support vector machine (SVM)	Internal	CV	accuracy: 88%
Wu et al. (2016) [75]	BD-E	BD-E = 21, demographically matched C = 21	computerized Cambridge Neurocognitive Test Automated Battery	least absolute shrinkage selection operator (LASSO)	Internal	leave-one -out CV	accuracy: 71%, AUC: 0.71

C: (healthy) control; BD: Bipolar Disorder; SZ: Schizophrenia; CV: Cross-Validation; AUC: Area Under the ROC Curve.

**Table 3:** CAD methods for mental and neurological disorders (cont’d).

References	Goal	Sample Size	Data	Classifier	Internal, External, Validation	Type of Validation	Index (the Best Result)
Sutcubasi et al. (2019) [76]	BD and SZ	BD = 41, SZ = 39, C = 23	MRI	artificial neural network (ANN)	Internal	4-fold CV	accuracy: 81.25%
Zeng et al. (2018) [77]	Multi-Site Diagnostic Classification of SZ	7 sites: patients = 357, C = 377	fMRI	Discriminant Autoencoder Network with Sparsity constraint (DANS)-SVM	Internal	10-fold CV leave-site-out	Accuracies of 85% and 81% in multi-site pooling classification and leave-site-out transfer classification
Oh et al. (2020) [78]	SZ	Internal: SZ = 443, C = 423 External: SZ = 30, C = 30	MRI	three-dimensional convolutional neural network (3DCNN)	Both	10-fold CV	External: accuracy rate: 70%, AUC: 0.72 internal: AUC: 0.959 overall accuracy rate: 97%
Aslan et al. (2020) [79]	SZ	two separate sets of data (children and adult) Dataset A: C = 39 SZ = 45, Dataset B: C = 14 SZ = 14	EEG 16/19 electrode	Convolutional Neural Network architecture (VGG-16)	Internal	Holdout (80% train, 20% test)	accuracy of 95% and 97% in two datasets AUC: 0.95 and 0.974
Shalbaf et al. (2020) [80]	SZ	C = 14 SZ = 14	EEG 19 channel	ResNet-18-SVM	Internal	10-fold CV	accuracy: 99%
Naira et al. (2019) [81]	SZ and C	C = 39 SZ = 45	EEG 16 channel	CNN	Internal	Holdout (96% train, 4% test)	Accuracy: 90%
Rozycki et al. (2018) [82]	SZ	5 sites: (941 adult participants, SZ = 440	MRI	SVM	Internal	10-fold CV & leave-site-out	cross-validated prediction accuracy: 76% (AUC: 0.84) leave-site-out validation accuracy/AUC range of 72–77%/0.73–0.91
Shim et al. (2016) [83]	SZ	SZ = 34 HC = 34	EEG 62 electrode	support vector machine (SVM)	Internal	leave-one-out CV	accuracy: 88%

C: (healthy) control; BD: Bipolar Disorder; SZ: Schizophrenia; MDD: Major Depressive Disorder; CV: Cross-Validation; AUC: Area Under the ROC Curve.

**Table 4:** CAD methods for mental and neurological disorders (cont’d).

References	Goal	Sample Size	Data	Classifier	Internal, External, Validation	Type of Validation	Index (the Best Result)
Winterburn et al. (2019) [84]	SZ and C	435 subjects total	MRI	support vector machines (SVM)	Internal	10-fold CV, and a held-out (2:1 ratio)	accuracy: 74%
Lin et al. (2018) [85]	SZ	SZ = 89 HC = 60	Blood sample	naive Bayes model	Internal	10-fold CV	AUC = 0.94
Cai et al. (2020) [86]	SZ	Internal: SZ = 51 HC = 51 External: SZ = 34 HC = 27	rs-fMRI	linear discriminant analysis (LDA)	Both	Leave one out CV	Accuracy Internal: 0.725, External: 0.70
Qureshi et al. (2017) [87]	SZ	Normal control = 72 SZ = 72	rs-fMRI & sMRI	Extreme Learning Machine Classifier (ELM)	Internal	10-by-10-fold CV	Train accuracy = 0.99 Test accuracy: 0.99
Juneja et al. (2018) [88]	SZ	D1: C = 34, SZ = 34 D2: C = 25, SZ = 25	fMRI	SVM	Internal	Leave one out CV	Accuracy: D1: 97% D2: 98%
de Boer (2020) [89]	SZ	SZ = 26, C = 22	Subject speech	logistic regression model	-	-	AUC: 0.86
Oh et al. (2019) [90]	Automated Diagnosis of SZ	C = 14 SZ = 14	EEG 19 electrode	CNN (Convolutional Neural Network)	Internal	non-subject based testing (10-fold CV) and subject-based testing (14-fold CV)	accuracy of 98% for non-subject-based testing, accuracy of 81%, for subject-based testing
Santos-Mayo et al. (2017) [91]	SZ	SZ = 16 C = 31	EEG 17 electrode	SVM & Multilayer Perceptron (MLP)	Internal	Holdout	AUC: 0.96 (Total-15HzJ5-MLP&SVM), AUC: 0.98, Right Hemisphere35Hz-J5-SVM)
Chatterjee (2018) et al. [92]	SZ	D1: SZ = 30, C = 30 D2: SZ = 25, C = 25	fMRI	SVM	Internal	Leave-One-Out CV	Mean classification accuracy D1 99.5% D2 97.4%

C: (healthy) control; SZ: Schizophrenia; CV: Cross-Validation; AUC: Area Under the ROC Curve.

**Table 5:** CAD methods for mental and neurological disorders (cont’d).

References	Goal	Sample Size	Data	Classifier	Internal, External, Validation	Type of Validation	Index (the Best Result)
Ding et al. (2020) [93]	depression recognition (Depression and Normal)	Depression-prone = 108 C = 585	Internet behavior characteristics of Internet users on social media	DISVM (deep integrated support vector Machine)	Internal	Holdout	accuracy P (Precision) = 86%
Li et al. (2019) [94]	Mild depression	depressed = 24 C = 27	EEG signals (Number of channels = 128)	convolutional neural network (CNN)	Internal	24-fold CV	accuracy = 86%
Byeon et al. (2017) [95]	Depression	of 9024, subjects (depression = 2627)	general characteristics, economic level, employment, health, and health care, and marriage	Chi-Squared Automatic Interaction Detection (CHAID)	Internal	10-fold CV	predictive accuracy of the model was 74%,
Kautzky et al. (2017) [96]	Depression	C = 62 acutely depressed = 19	PET using the radioligand [carbonyl-11C]	randomForest (RF) and (SVM)	Internal	10-fold CV	RF reached an accuracy around 0.725 for all samples (vs 0.750 for SVM)
Lin et al. (2018) [97]	Predict MCI-to-AD conversion	188 AD, 229 NC, and 401 MCI subjects	MRI data	convolutional neural networks (CNN) extreme learning machine	Internal	leave-one-out CV	accuracy: 80% , AUC: 0.86
Ding et al. (2018) [98]	AD classification (Normal, Very mild AD, Mild AD, Moderate AD)	861 participants in the non-imaging dataset 613 participants in brain imaging (MRI) dataset, 207 participants in PET data	Demographics, medical history, ApoE genotype, psychological/functional assessments, blood analyses, and clinical diagnoses. brain imaging dataset (structural MRI and PET data)	Bayesian network (BN)	Internal	Holdout (90% 10-fold CV, 10% test), Resampling: Synthetic minority oversampling technique (SMOTE)	AUC: 0.91

C: (healthy) control; SZ: Schizophrenia; CV: Cross-Validation; AUC: Area Under the ROC Curve.

**Table 6:** CAD methods for mental and neurological disorders (cont’d).

References	Goal	Sample Size	Data	Classifier	Internal, External, Validation	Type of Validation	Index (the Best Result)
Lu et al. (2018) [99]	Early Diagnosis of AD ((Normal controls (NC), stable MCI (sMCI), the progressive MCI (pMCI), AD clinically diagnosed	1051 subjects NC = 304 sMCI = 409 pMCI = 112 probable AD = 226	FDG-PET images and structural MRI	ensemble multiple classifiers (Multiscale Deep Neural Networks)	Internal	Holdout (10-fold CV in training)	Accuracy: 94%, NC vs. AD, Accuracy: 82%, sMCI vs. pMCI and Accuracy: 83%, sMCI vs. pMCI with sample from NC & AD
Fiscon et al. (2018) [100]	Classifying AD (AD,MCI,C)	C = 23, MCI = 37, AD = 49	multi-channel EEG signals 19 electrode	Decision Trees classifiers	Internal	leave-one-out CV	Accuracy: 92% for HC vs. MCI, Accuracy: 83% for HC vs. AD, Accuracy: 73% for HC vs. CASE (MCI+AD), and accuracy: 79% for MCI vs. AD
Aidos et al. (2017) [101]	Predicting AD (Cl, MCI, and AD)	58 subjects for AD with four images each, 88 subjects with MCI with six images each, and 60 subjects for CN with five images each	FDG-PET scans	SVM with an RBF kernel	Internal	Repeated Holdout-20 times (70% training 10-fold CV, 30% test)	in a multiclass classification task, 59% accuracy at baseline and goes up to 69% in the follow-up

C: (healthy) control; AD: Alzheimer disease; MCI: mild cognitive impairment; CV: Cross-Validation; AUC: Area Under the ROC Curve.

**Table 7:** CAD methods for mental and neurological disorders (cont’d).

References	Goal	Sample Size	Data	Classifier	Internal, External, Validation	Type of Validation	Index (the Best Result)
Bin-Hezam et al. (2019) [102]	Detecting Dementia based on risk factors	1812 subjects	Demographics and Medical History	logistic regression & random forest	Internal	Holdout (StratifiedKFold 75% train, 25% test) and 10-Fold CV	Accuracy: 91.53%, (dementia vs. non-dementia), Accuracy: 77%, (multi-class prediction (CN vs. MCI vs. dementia)
Zhou et al. (2016) [103]	Predictors of hospital admission of patients with dementia Health and dementia	59,298 dementia patients (30,178 were admitted to hospital and 29,120 remained with GP care)	initial GP read codes, diagnostic events, five medication events, three procedural events, sex, age	neural network with entropy regularization	Internal	10-fold cross-validation	AUC: 0.76
Choi et al. (2018) [104]	A diagnostic framework for dementia (normal vs. dementia)	2666 cognitively normal elderly = 2666 dementia patients = 435	Mini-Mental Status Examination (MMSE) as a screening test, KLOSCAD-N assessment	deep neural networks (DNNs)	Internal	Holdout (80% training (5-fold CV), 20% test)	Accuracy of 93%,
Moreira et al. (2016) [105]	Diagnosis of patients with clinical suspicion of dementia	AD = 209 MCI = 97, Others = 218)	Demographic, clinical, and screening tests	J48 (decision tree algorithm C4.5)	Internal	Holdout (75% train, 25% test) Resampling: SMOTE just for MCI	AD Accuracy:80%, AUC: 0.849, MCI Accuracy:91%
Bang et al. (2017) [106]	Dementia diagnosis normal groups and dementia groups	14,917 participants	Clinical data called CREDOS	Support Vector Machine (SVM)	Internal	Holdout (40% for training, 30% for test and 30% for validation)	AUC: 0.96 Accuracy: 90%

C: (healthy) control; AD: Alzheimer disease; MCI: mild cognitive impairment; CV: Cross-Validation; AUC: Area Under the ROC Curve.

**Table 8:** CAD methods for suicide prediction.

References	Goal	Sample Size	Data	Classifier	Internal, External, Validation	Type of Validation	Index (the Best Result)
Walsh et al. (2017) [107]	suicide	5167 adult patients 3250 patients made a suicide attempt (cases), and 1917 controls	(a) demographic data (b) diagnoses based on claims data (c) past health care utilization (d) evidence of prior suicide attempts (e) body mass index (f) socioeconomic status (g) medication data	Random forests	Internal	boot strapping (rep = 100)	AUC: 0.84
Walsh et al. (2018) [108]	Suicide	496 adolescents with other self-injury (OSI), 7059 adolescents with depressive symptoms, and 25,081 controls	longitudinal clinical data in adults: diagnostic, demographic, medication, and socioeconomic factors	random forests	Internal	boot strapping	OSI C (AUC = 0.83) at 720 days; AUC = 0.85 at 7 days) and depressed C (AUC = 0.87), depressed C (AUC = 0.87) and 0.90 at 720 days at 7 days) General hospital C (AUC 0.94 at 1720 days, 0.97 at 7 days).
Just et al. (2017) [40]	suicidal ideation	Internal: (17 suicidal ideation versus 17 Controls External: 21 suicidal ideation	fMRI	Gaussian Naive Bayes (GNB)	both	leave out half of the participants from each group	suicidal vs. C accuracy of 0.91, those had previously attempted those who had not (accuracy of 0.94) External: suicidal ideation from C accuracy of 0.87
Cheng et al. (2017) [109]	Suicide Risk assessment	974 Weibo users	Weibo posts	Support Vector Machine (SVM)	internal	leave-one-out	AUC: 0.6
Oh et al. (2017) [110]	Suicide	Patients with depression and anxiety disorders (*n* = 573)	31 psychiatric scales and 10 sociodemographic elements	artificial neural network	Internal	Hold out	(1-month) accuracy: 93.7%., AUC: 0.93, (1-year): 90.8%, AUC: 0.87, (lifetime) Accuracy: 87.4%, AUC: 0.89
Hettige et al. (2017) [111]	Suicide attempters in schizophrenia	345 participants	clinical, demographic, and sociocultural	Regularized logistic regression	internal	Stratified 10-fold CV	accuracy: 67% AUC: 0.71

C: Control; CV: Cross-Validation; AUC: Area Under the ROC Curve.

**Table 9:** CAD methods for suicide prediction (cont’d).

References	Goal	Sample Size	Data	Classifier	Internal, External, Validation	Type of Validation	Index (the Best Result)
Ryu et al. (2018) [112]	Suicide	11,628 individuals (5814 suicide)	Korea National Health and Nutrition Examination Survey (KNHANES)	random forest	internal	Hold out (training: 10-fold CV)	AUC = 0.85 accuracy of 0.821
Jung et al. (2019) [113]	adolescents of high-risk suicide	*n* = 59,984 (7443 adolescents with a history of suicide)	Korean Young Risk Behavior Web-based Survey (KYRBWS)	extreme gradient boosting (XGB)	internal	5-fold CV	Accuracy:79% AUC = 0.86
Lin et al. (2020) [114]	Suicide	3546 military men and women	The questionnaire for the military personnel composed of five psychopathological domains, anxiety, depression, hostility, interpersonal sensitivity and insomnia)	SVM And multilayer perceptron	internal	10-fold CV	Accuracy:100% AUC:100%
Su et al. (2020) [115]	Suicide in children and adolescents	Suicide-positive subjects (*n* = 180) Suicide-negative subjects (*n* = 41,541)	Longitudinal clinical records demographics, diagnosis, laboratory tests, and medications	logistic regression	internal	Repeated Hold out: 10 times (90% training)	AUC: 0.86
Choi et al. (2018) [116]	Suicide	819,951 subjects Suicidal death No (*n* = 817,405) Yes (*n* = 2546)	qualification and medical services claim data	Cox regression, SVM and deep neural networks (DNNs)	internal	Hold out (70% training, 30% validation)	AUC of Cox regression: 0.688, of SVM: 0.687, of DNN 0.683
Chen et al. (2020) [117]	Suicide	541,300 inpatient	demographic characteristics, socioeconomic	ensemble learning of elastic net penalized logistic regression, random forest, gradient boosting, and a neural network	internal	Hold out (80% training, 20% test) (training: 10-fold CV)	AUC = 0.88

C: Control; CV: Cross-Validation; AUC: Area Under the ROC Curve.

**Table 10:** CAD methods for suicide prediction (cont’d).

References	Goal	Sample Size	Data	Classifier	Internal, External, Validation	Type of Validation	Index (the Best Result)
Edgcomb et al. (2021) [118]	Differentiate Risk of Suicide Attempt and Self-harm	1628 women (University of California Los Angeles) 140,848 women (New York City Clinical Data Research Network)	Sociodemographic data, medications, health care utilization, and diagnostic codes	decision tree	internal	10-fold CV	University of California Los Angeles (Accuracy: 84%, AUC: 0.73) New York City Clinical Data Research Network (Accuracy: 84%, AUC: 0.71)
Agne et al. (2020) [41]	suicide attempt in patients with obsessive-compulsive disorder	959 outpatients with OCD	clinical and sociodemographic variables	elastic net	internal	Hold out (75% training, 25% test) (10-fold CV in training)	AUC: 0.95 accuracy: 85.97%
Haroz et al. (2020) [119]	Identify patients with the highest risk for suicide	*n* = 2390 individuals	demographics, educational history, past mental health, and substance use	regularized regression using ridge regression	internal	Hold out (train, test: two-thirds/one-third split)	AUC = 0.87
Ryu et al. (2019) [120]	Suicide	5773 subjects	Korea National Health and Nutrition Examination Survey (KNHANES)	random forest	internal	Hold out (Train 70%, test 30%) (training 10-fold CV)	AUC = 0.947 accuracy: 0.889
Miché et al. (2020) [121]	Suicide	*n* = 2797 adolescents and young adults aged 14–24 years	demographics, cognitive abilities, family history of psychopathology, general psychopathology, psychosis, prior self-injurious thoughts or behaviors, social factors, and treatment history	logistic regression, lasso, ridge, and random forest	internal	repeated nested 10-fold CV	mean AUCs of logistic regression, lasso, ridge, and random forest, were 0.828, 0.826, 0.829, and 0.824, respectively
Shen et al. (2020) [122]	Suicide	4882 medical students	Self-report data on sociodemographic and clinical characteristics were collected online via the website or through the widely used social media app, WeChat	random forest	internal	5-fold CV	(AUC) = 0.9255 Accuracy: 90.1%

C: Control; CV: Cross-Validation; AUC: Area Under the ROC Curve.

**Table 11:** CAD methods for suicide prediction (cont’d).

References	Goal	Sample Size	Data	Classifier	Internal, External, validation	Type of Validation	Index (the Best Result)
Parghi et al. (2020) [123]	near-term suicidal behavior	*n* = 591, attempted: *n* = 20, those who did not (*n* = 571)	Suicide Crisis Inventory (SCI) data, which measures the Suicide Crisis Syndrome, a presuicidal mental state	gradient boosting	internal	enhanced bootstrap	Accuracy 0.981
Naghavi et al. (2020) [42]	Suicide	573 university students	Different types of Questionnaire	decision trees	internal	3-fold CV	AUC = 0.90
Cohen et al. (2020) [124]	Suicide	Internal (ACT Study, STM Study) External (267 interviews, 60 students, 29 students indicating suicide or self-harm risk)	language samples, depression, and standardized suicidality scale scores, and therapist impression of the client’s mental state	extreme gradient boosting	both	Leave-one-site-out	AUC: 0.78
Zheng et al. (2020) [125]	Suicide	The retrospective cohort (118,252 individuals, cases: 255) The validation cohort (118,095 individuals, cases: 203)	Electronic health records (EHRs)	Deep neural network	both	CV	AUC: 0.77
Tadesse et al. (2020) [126]	Suicide Ideation in Social Media Forums	3549 suicide-indicative posts, 3652 non-suicidal posts	Reddit social media dataset	LSTM-CNN Long Short-Term Memory Convolutional Neural Network	Internal	CV	Accuracy: 93.8

C: Control; CV: Cross-Validation; AUC: Area Under the ROC Curve.
